# The effects of sodium butyrate and inulin supplementation on angiotensin signaling pathway via promotion of *Akkermansia muciniphila* abundance in type 2 diabetes; A randomized, double-blind, placebo-controlled trial

**DOI:** 10.15171/jcvtr.2017.32

**Published:** 2017-11-25

**Authors:** Neda Roshanravan, Reza Mahdavi, Effat Alizadeh, Abed Ghavami, Yalda Rahbar Saadat, Naimeh Mesri Alamdari, Shahriar Alipour, Mohammad Reza Dastouri, Alireza Ostadrahimi

**Affiliations:** ^1^Nutrition Research Center, Students Research Committee, School of Nutrition, Tabriz University of Medical Sciences, Tabriz, Iran; ^2^Nutrition Research Center, School of Nutrition, Tabriz University of Medical Sciences, Tabriz, Iran; ^3^Department of Medical Biotechnology, Faculty of Advanced Medical Sciences, Tabriz University of Medical Sciences, Tabriz, Iran; ^4^Department of Nutrition, School of Nutrition, Tabriz University of Medical Sciences, Tabriz, Iran; ^5^Student Research Committee, School of Health, Iran University of Medical Sciences, Tehran, Iran; ^6^Department of Molecular Medicine, Nutrition Research Center, Tabriz University of Medical Sciences, Tabriz, Iran; ^7^Biotechnology Institute Central Laboratory, Biotechnology and Stem Cell Institute, Ankara University, Ankara, Turkey

**Keywords:** Inulin, Sodium Butyrate, TNF-α, *Akkermansia muciniphila*, Diabetes, Angiotensin Signaling Pathway

## Abstract

***Introduction:*** Inflammation has a crucial role in the progression of cardiovascular disease in diabetes. Tumour necrosis factor-α (TNF-α) as an inflammatory marker induces angiotensin II (Ang II) related hypertension pathway in diabetic patients. Gut modulation via prebiotics may ameliorate hypertension caused by inflammation. The aim of this study was to investigate the role of sodium butyrate (NaBut) and inulin supplements on inflammatory and oxidative stress parameters in type 2 diabetic patients.

***Methods:*** In this clinical trial, 60 overweight and obese diabetic patients were recruited and randomly allocated into four groups. The groups received, respectively, 600 mg/d NaBut (group A), 10 g/d inulin powder (group B), both inulin and NaBut (group C), or placebo (group D) for 45 consecutive days. Blood and stool samples were collected at baseline and after intervention. Quantitative real-time PCR analysis targeting the 16S rRNA gene of *Akkermansia muciniphila* was done. We assessed the TNF-α mRNA expression and the serum levels of the high sensitive C-reactive protein (hs-CRP) and malondialdehyde (MDA).

***Results:*** There was a significant increase in *A. muciniphila* percent change in inulin and butyrate supplemented groups (*P* < 0.05). Furthermore, significant decrease was seen in TNF-α mRNA expression in group A (fold change 0.88 ± 0.16, *P*< 0.05), group B (fold change 0.75 ± 0.18, *P *< 0.05) and group C (fold change 0.91 ± 0.32, *P *< 0.05). Also hs-CRP, MDA and diastolic blood pressure levels decreased significantly in these groups (*P *< 0.05).

***Conclusion:*** Intervention had significant effects on inflammatory and oxidative stress parameters and led to improvement of hypertension. However, further investigations are needed to make concise conclusions.

## Introduction

The altered composition of intestinal microbiota have been linked to a number of metabolic diseases—including obesity, cancer, cardiovascular diseases, and diabete.^1^ Recently, numerous studies suggested a possible involvement of gut microbiota dysbioses in the development of insulin resistance, type 2 diabetes (T2D) and its complications.^2^ Cardiovascular disease (CVD) is one of the main causes of death in developing countries. Hypertension (high blood pressure), obesity and diabetes are dominant risk factors known to affect cardiovascular health.^3^

Angiotensin II (Ang II) is a systemic vasoconstrictor and recently its well-known role has been established in the pathophysiology of T2D. According to clinical studies, blocking Ang II modulated diabetes cardiac problems. To date, it has been reported that gut microbiota contributes with AngII‐induced hypertension and metabolic inflammation.^4,5^

Most pioneering studies have found that *Akkermansia muciniphila,* which is related to the genera *Prosthecobacter* and *Verrucomicrobium,* is the gram-negative, strictly anaerobic bacterium of this phylum that is colonized in the human intestinal tract and is known as mucin-associated bacteria.^6^ Animal-based studies have reported anti-inflammatory properties of this­ bacterium.^7,8^ Its modulation of inflammatory response is thought to be due to genes expression pathways.^8,9^

Tumour necrosis factor-α (TNF-α) is a proinflammatory cytokine which involved in diverse physiological processes, such as inflammation, cell growth and apoptosis. Previous investigations proved that inflammation is a crucial component in the pathogenesis of hypertension and CVD. The cross-talk between Ang II and TNF-*α* may play an important role in the modulation of blood pressure in diabetic patients. Additionally, Ang II increased oxidative stress by stimulation of Nuclear Factor-kappa B (NF-κB) which is one of the well- known redox sensitive transcription factors.^10^

A growing body of evidence showed that systemic inflammation diminished the activity of antioxidant enzymes and total antioxidant capacity.^11^ High sensitive C-reactive protein (hs-CRP) and malondialdehyde (MDA) are markers of systemic inflammation and oxidative stress particularly in subjects with diabetes and CVD.^12^ Hs-CRP is a new biomarker for predicting the risk of cardiovascular events. Animal based study demonstrated that blockade of TNF-α can prevent the development of oxidative stress.^13^ Nevertheless, the underlying mechanisms involved remain unclear.

Prebiotics according to the definition given by the Food and Agriculture Organization (FAO), are “non-viable food component that confers a health benefit on the host associated with modulation of the microbiota”.^14^ These low-digestible carbohydrates with known anti-inflammatory properties ferment in the colon into short-chain fatty acids (SCFA: e.g. butyrate, propionate, and acetate).^15^ The known prebiotic, inulin, is composed of multiple fructose units with a terminal glucose and belongs to a class of compounds known as fructans.^16^ High-performance (HP) inulin has a long-chain, high-molecular weight mix of inulin-type fructans, not including any fructan that has a degree of polymerization <10.^4^ Based on several studies, prebiotics has specific effects on inflammation and metabolic endotoxemia via changes in gut microbiota and fermentation products.

Previous studies claimed that butyrate as a “postbiotics” product—may have gastrointestinal protective effects. This anti- inflammatory agent can participate in the control of various metabolic processes by modulation of host genes expression.^17^

Based on this evidence; we aim to investigate the effects of sodium butyrate (NaBut) and HP inulin supplementation on Ang signaling pathway via promotion of gut bacterium *A. muciniphila* growth and alteration in *TNF-α* mRNA expression in T2D patients.

## Materials and Methods

### Study population

Sixty subjects with T2D took part voluntarily in this randomized, double-blind, placebo-controlled clinical trial. All the patients were recruited from the AZAR Cohort Study (Persian cohort, http://persiancohort.com) and Rohzendeh Health and Therapeutic Center in Shabestar (East Azerbaijan, Iran). The study was performed between August and December 2016. The criteria for the diagnosis of diabetes were taken into account based on the American Diabetes Association criteria: fasting glucose ≥126 mg/dL or hemoglobin A1c ≥6.5%.^23^ Inclusion criteria for the participants were: a history of DM >6 months, consumption of anti-diabetic drugs, age range of 30–55 years, and body mass index (BMI) of 27–35 kg/m^2^. The specified exclusion criteria were a history of diagnosed gastrointestinal disease, coronary heart disease, renal failure, thyroid disease, liver or pancreatic illness, pregnancy or lactation, insulin therapy, consumption of pre-or probiotics, antibiotic or antacid drugs, and alcohol or tobacco use at the time of recruitment. The usage of first line preventative drugs for the treatment of diabetes, hydroxy methyl glutaryl-coenzyme-A reductase inhibitors (statins) for the treatment of hypercholesterolemia, and ACE inhibitors for hypertension were not of those exclusion criterion.

The sample size of the study was determined on the basis of prior data with confidence interval 95% and power 90%, based on fasting blood sugar parameter.^24^ This calculation determined a total sample size of 52 individuals, plus eight people in case of withdrawals. Hence, a total of 60 patients were needed. These 60 patients were distributed randomly (with allocation ratio 1:1) using randomized block procedure to one of four treatment orders (A, B, C, or D) by a computer-generated allocation schedule (Random Allocation Software) in which A was specified as the butyrate group, B was given to the inulin group, C was the inulin + butyrate group, and D was appointed as the placebo group. Fifteen patients were in each group. Group A took 6 capsules of 100 mg NaBut (BodyBio, USA) and 10 g/d of starch powder as placebo. Each capsule was consumed before and after each meal 6 times per day as recommended by the manufacturer. Ten grams of inulin powder was divided as 2 sachets containing five grams in each. Group B consumed 10 g/d of HP inulin supplement powder (Sensus, Borchwef 3, 4704 RG Roosendaal the Netherlands) as well as six starch capsules as placebo. Group C was appointed to the concomitant use of NaBut capsules and HP inulin powder (10 g of inulin powder + six capsules of sodium butyrate), while group D (control group) received six 100 mg starch capsules as well as 10 g of starch powder as placebo which were matched to study supplements in terms of color and size. All the participants consumed *allocated supplements* for 45 consecutive days. A local physician carried out randomization of the participants between groups. After the provision of supplements, the physician had no relationship with the study patients or involvement in other study processes. Based on consumed medical drugs (glucose lowering and anti- hyperlipidemia drugs) and disease duration all the patients were classified in the study. All of the patients were inquired to maintain their previous habitual lifestyle, physical activity, and dietary intake during the intervention. A health center staff member called on each patient weekly to minimize their withdrawal and certify the consumption of the supplements. All subjects were visited every 15 days during the trial period.

### DNA isolation from stool samples

The *stools* specimens are *collected* pre and post intervention and were stored at -80^◦^C for bacterium analysis. DNA was extracted using a fecal DNA isolation kit (BioBasic, Canada) based on the manufacture’s protocols. QRT-PCR (quantitative real-time PCR) is used to determine faecal concentration of *A. muciniphila.* The primers were designed from the variable regions of the 16S rRNA gene sequences of this bacterium from NCBI GenBank program based on the following sequences: F- CAACGCTTGAGACCTCTGTATT and R- CCTGTCATGTGGGAGCAAATTA. PCR amplification and detection were quantified using real- time PCR (Mic –qPCR, Australia). The cycle threshold (CT) for each sample was compared with a standard curve made by diluted genomic DNA of the *A. muciniphila* type strain (ATCC BAA-835). We determined logarithmic relationships between the mean CT and *A. muciniphila* colony forming unit (CFU). Date was expressed based on natural logarithm (log_e_ CFU)/100 mg of fecal samples. Each assay performed in triplicate.

### Isolation of peripheral blood mononuclear cells

Five milliliter venous blood samples were collected in fasting status from all of the participants to vacuum collection tubes containing EDTA (Vacutainer K2E) at baseline and after the intervention. Peripheral blood mononuclear cells (PBMCs) were isolated through density gradient centrifugation using Ficoll-Histopaque solution gradient (ficoll- paque, GmbH). Total RNA purification was extracted using ambion Trizol LS reagent (Thermo Fisher Scientific, USA), according to the manufacturers’ protocol.

### RNA isolation protocol for TNF-α gene

The RNA quality and quantity were assessed using the NanoDrop spectrophotometer (NanoDrop One/One^c^, Thermo Scientific) after extraction of total RNA. Total RNA was converted to complementary DNA (cDNA) using total isolated RNA, random hexamer primer and reverse transcriptase according to manufacturer’s protocol (Thermo Scientific RevertAid First Strand cDNA Synthesis Kit, USA).

### Real-time PCR for gene

The level of TNF-α mRNA was examined by SYBR Green Master mix (Thermo Fisher Scientific, USA). The primer sequences were designed by using PrimerBank. Data were normalized to β-actin expression by the ΔΔC_T_ comparative method.^18^ All samples were run in triplicate. Fold change of the TNF-α mRNA was calculated as relative expression post intervention/control one.

### Hs-CRP and MDA measurement

Blood samples were collected before and after intervention for an overnight fasting of 12 hours and serum samples were stored at − 80°C until analyses. MDA levels were measured by the thiobarbituric acid reaction with acid, which was extracted with *n*-butanol, and measured spectrophotometrically at a wavelength of 523 nm. High sensitivity C-reactive protein concentration (hsCRP) was assessed by latex-particle-enhanced immunoturbidometric assay.

### Blood pressure measurements

For each patient blood pressure was measured in the same arm twice using mercury sphygmomanometer after the individual was seated at rest 10–15 minutes. The systolic and diastolic measurements represent the mean of two readings.

### Statistical analysis

Statistical assay was executed by SPSS statistical software (SPSS Inc., Chicago, IL, USA, version 23). One-sample Kolmogorov-Smirnov test was used to assay the normality of data. All the results are expressed as mean ±SD. Differences in group means at baseline characteristics were revealed using one-way ANOVA. Comparison of data at baseline characteristics and end of the study was performed using paired *t* test.

The one-sample ***t*** test was used for comparisons between internal control and after intervention values for gene expression. The data were analyzed by one-way ANOVA followed by a *post-hoc* Tukey test for multiple comparisons between means. An analysis of covariance test (ANCOVA) was done to adjust the effects of confounding factors. The percent change of variables was calculated by using the formula: [(after intervention values – baseline values)/baseline values] × 100. *P* values less than 0.05 were considered to be significant. The Prism software, version 6.0 (GraphPad, CA, USA) was used for figure drawing.

## Results

**Figure 1:**
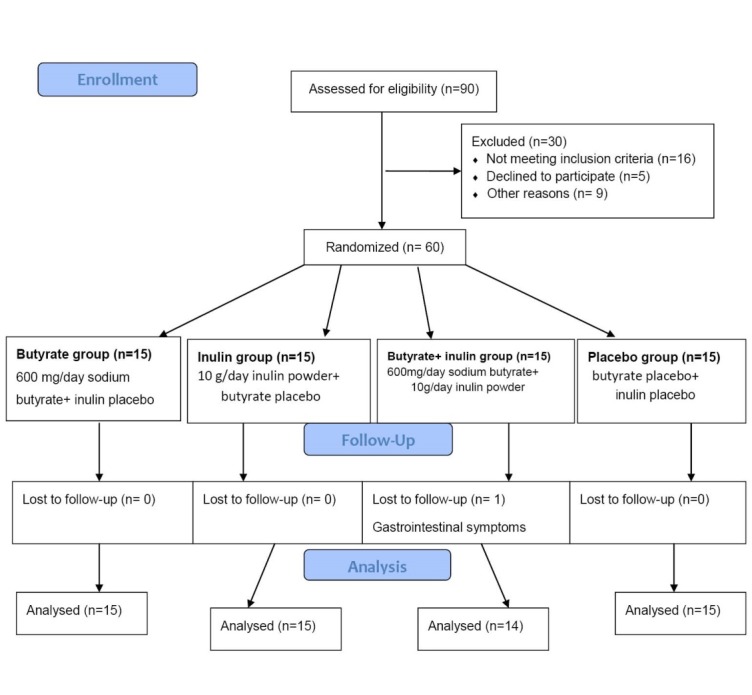
Flowchart of design and protocol of the study.

Figure 1 shows the flowchart of this trial. Of 60 selected patients for this study fifty-nine participants completed the trial and were included in the statistical analysis. One subject in group C withdrew from the study because of severe gastrointestinal symptom. As seen in Table 1, none of the baseline characteristics showed any statistically significant differences between the 4 study groups with the exception of diastolic blood pressure.

**Table 1:** Baseline characteristics of the study population

**Variables**		**The study groups**				***P***
		**Butyrate (n=15)**	**Inulin (n=15)**	**Butyrate + Inulin (n=14)**	**Placebo (n=15)**	
Gender	Male	5 (33.3)	8 (53.3)	4 (28.6)	5 (33.3)	0.667*
	Female	10 (66.7)	7 (43.7)	10 (71.4)	10 (66.7)	
Age (y)		45.87± 8.05	51.47± 6.46	47.14± 7.99	51.73± 8.44	0.099**
Weight (kg)		80.52± 12.00	86.07± 10.33	80.70± 13.62	81.74± 16.64	0.641**
Height (cm)		164.73± 11.44	168.33± 8.60	163.21±11.38	162.60± 9.81	0.441**
BMI (kg/m^2^)		29.80± 4.52	30.37± 2.82	30.31± 4.25	30.86± 5.41	0.931**
Systolic Blood pressure (mm Hg)		135.33± 16.19	139.00± 24.23	123.93± 18.93	126.67± 20.23	0.126*
Diastolic Blood pressure (mm Hg)		85.67± 8.42	82.33± 8.63	77.64± 7.07	79.00± 6.60	0.032*
Disease duration (y)		1.63± 0.36	1.61± 0.34	1.71± 0.40	1.43± 0.31	0.229**
Baseline FBS (mg/dL)		144.73± 40.55	167.07± 82.17	176.86± 56.10	129.53± 26.38	0.099**
Metformin 500 mg (tablets/day)		2.66± 1.21	2.70± 0.90	2.85± 1.08	2.28± 0.89	0.101**
Glibenclamide 5 mg (tablets/day)		1.19± 0.73	1.35± 0.94	0.92± 0.60	1.3± 0.65	0.221**

BMI, body mass index, FBS: fasting blood sugar. *P value was reported based on chi-square test. **P value was reported based on ANOVA. Quantitative and qualitative variables were represented as frequency (percent) and Mean± SD respectively. *p*<0.05 statistically significant.

As shown in Table 2 the percentage changes of *A. muciniphila* abundance indicated a significant increase in group A and B in comparison with the placebo group (*P* < 0.05). Interestingly, a non significant rise in this bacterium concentration was seen after supplementation in group C. The post hoc Tukey tests were used for two-by-two comparisons, after adjusting for confounding variables the results showed significant differences in group A and B compared with group D (*P* < 0.05).

**Table 2:** Colony forming units of *Akkermansia muciniphila*, inflammatory and oxidative stress parameters before and after intervention between and within groups

**Variable**		**The study groups**				***P*** ** value** ^**^
		**Butyrate (n=15)**	**Inulin (n=15)**	**Butyrate+ Inulin (n=14)**	**Placebo (n=15)**	
LnCFU *Akkermansia muciniphila*	Before	8.51±2.84	7.26±3.67	8.60±3.38	8.57±2.42	0.013
	After	10.81±3.43	9.94±3.35	10.05±2.52	8.95±2.94	
	*P* ^*^	0.001	0.006	0.160	0.210	
	%‏ Change	27.29 (13.72-61.39)	39.92 (4.99-124.79)	30.07 (-39.73-82.64)	21.48 (-31.80-51.52)	
hsCRP (mg/L)	Before	4.33±1.11	5.45±2.28	3.89±1.14	5.40±2.01	<0.001
	After	2.98±1.02	3.80±1.38	2.44±1.01	5.91±2.15	
	*P* ^*^	<0.001	0.005	0.001	0.027	
	% Change	-31.04 (-57.60-4.75)	-25.63 (-69.79-44.59)	-34.25 (72.53-10.50)	14.19 (18.60-100)	
MDA (nmol/mL)	Before	5.35±1.69	6.40±2.09	6.68±2.27	5.11±2.02	<0.001
	After	3.94±1.58	6.13±1.93	5.51±2.17	5.39±1.57	
	*P* ^*^	<0.001	0.045	0.009	0.180	
	% Change	-25.74 (-56.76-0.67)	-3.39 (-17.51-11.01)	-12.31 (-34.95-13.11)	11.09 (-11.81-62.90)	

Abbreviations: CFU: colony forming units, hsCRP: high-sensitivity C-reactive protein, MDA: malondialdehyde. ^*^ *P* value reported based on paired t-test. ^**^P value reported based on ANCOVA (adjusted on baseline value, baseline BMI & diastolic blood pressure). *P<* 0.05 statistically significant.

Inflammatory and oxidative stress biomarkers are summarized in Table 2. The four groups did not show any significant differences in baseline inﬂammatory biomarkers with the exception of hs-CRP, After 45 days, mean serum MDA and hs-CRP levels significantly decreased in group A, B and C (*P* < 0.05) whilst the placebo group increased level.

**Figure 2:**
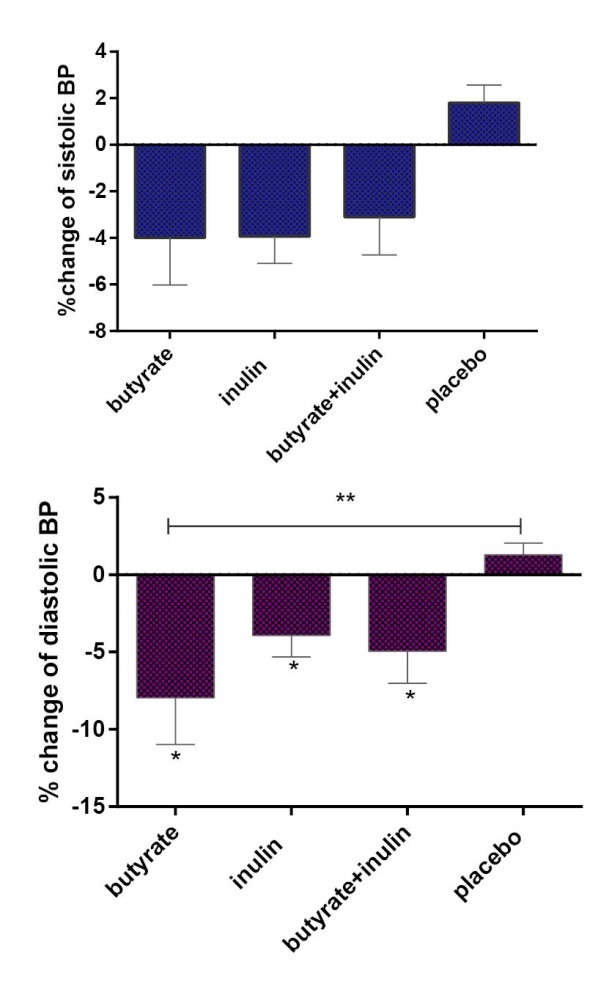
Comparison of percent change of blood pressure measurements between the study groups. *P<0.05 in within group analysis. **P<0.05 by ANCOVA (adjusted on baseline value). BP: blood pressure.

As shown in Figure 2, systolic blood pressure during the intervention remained unchanged (*P* > 0.05). But the between group comparison of diastolic blood pressure after adjusting for base line value indicated significant reduction in group A, B and C as compared with the placebo group (*P* < 0.05).

**Figure 3:**
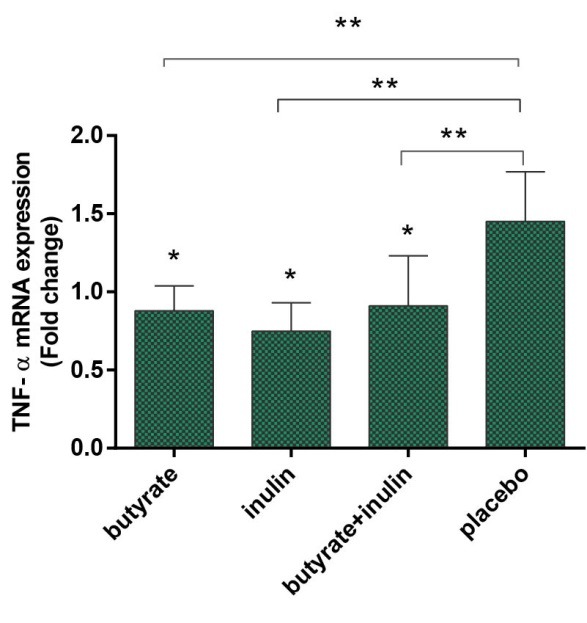
TNF-α mRNA expression in the study groups. Each point represented as mean ± SD. Data analysis was done using One Sample T test and ANOVA. Tukey was selected as post-hoc test. *P < 0.05 vs. control one. **P < 0.05 vs. Placebo. Tumour necrosis factor-α (TNF-α).

As presented in Figure 3, dietary supplementation of NaBut and HP inulin solo and simultaneously significantly decreased TNF-α fold change in 3 study groups (group A: fold change 0.88 ± 0.16, *P* < 0.05), (group B: fold change 0.75 ± 0.18, *P* < 0.05), (group C: fold change 0.91 ± 0.32, *P* < 0.05). Using ANOVA and Tukey *post hoc* test for two-by-two comparisons reported significant differences between group A, B and C in comparison with group* D* (*P*=0.032).

## Discussion

Several studies have demonstrated the crucial role of gut microbiome in improvement of host homeostasis and metabolism; anyhow, the molecular mechanisms of the crosstalk between gut *microbiote* and host for the control of metabolic disease such as CVD and diabetes are still unknown. T2D is specified by low-grade inflammation and oxidative stress, which cause chronic disturbances including hypertension, CVD and other metabolic disease.^19^ It is thought that some dietary supplements may act as a modulator of gut microbiota and therefore to be involved in inflammatory and metabolic conditions. We extend these findings to show the important effects of NaBut and HP inulin supplementation on the abundance of *A. muciniphila* bacterium and alteration in TNF-α mRNA expression in T2D patients. In addition, the effect of these dietary supplements on hs-CRP and MDA levels was assessed.

Several key findings emerge from this study: The abundance of *A. muciniphila* bacterium increased dramatically after butyrate and HP inulin supplementation, as well as, a non-significant increase in group C. Furthermore, dietary supplements significantly decreased the TNF-α mRNA expression. Other important findings from this study include a significant decreased in hs-CRP and MDA levels following intervention. In addition, there was a non-significant fall in systolic pressure but a significant decrease was seen in diastolic pressure with dietary supplements.

Recently a considerable body of evidence showed that inhibition of TNF-α expression can inhibit hypertension related vascular remodeling, inflammation and oxidative stress. Furthermore some researches proved that TNF-α protein is involved in Ang II signaling pathways, particularly by induced Ang II mediated hypertension. In addition, TNF-α also promotes Ang II-induced inflammation by initiation of oxidative stress.

In this sense, TNF-α has central role in initiating and promoting of inflammatory cascade. Additionally, its overexpression causes adverse cardiac remodeling. ^13^

However underlying molecular mechanisms remain not well understood. On the other hand, direct evidence for the possible involvement of gut microbiota dysbiosis in the development of CVD and some related pathways such Ang II signaling proposed a potential new therapeutic strategy for modulation of inflammation.^20^

In diabetes, changes in intestinal microbiota composition have been demonstrated previously. ^19^ In line with our findings, Everard et al^8^ demonstrated that prebiotic administration increases the fecal content of *A. muciniphila*. Here, we showed that *A. muciniphila* abundance could significantly increase following inulin and NaBut supplementation; however simultaneous consumption caused non-significant increase. This non intensified effect of taking inulin and butyrate supplementation at the same time may be due to unidentified mechanisms which were involved in this pathway.

Our results with regard to reductions in TNF-α are similar to some of previous studies.^21-24^ Despite these findings, contradictory results have also been reported.^25^ The diversity of results may be due to differences in type, dose, ethnicity, and genotype, duration of supplementation and basal levels of inﬂammatory status.

In agreement with several studies,^26,27^ we found beneficial effects of these supplementations on antioxidant status. Butyrate and HP inulin significantly reduced serum hs-CRP and MDA levels. Increased MDA and hs-CRP concentrations have been proved to be notably associated with cardiovascular risk factors such as hypertension.^28^ In line with this report we found significant reduction in diastolic blood pressure in the treatment groups as compared to the placebo group. Furthermore, a potent capacity in reducing systolic blood pressure was seen following intervention which may be improved with high dose and longer intervention period. Some studies have examined the effects of prebiotics supplementations on blood pressure levels and the diversity of effects have been reported.^29^

Several studies supported the anti- inflammatory role of prebiotics and butyrate previously.^30^ In vitro studies indicated that the most beneficial effects of butyrate can be attributed to its ability to inhibit the activation of the transcription factor Nuclear Factor-kappa B (NF-κB). NF-κB regulates many genes expression which involved in early inflammatory responses such as TNF-α, Ang II and CRP. Butyrate also improves the expression of the PPARγ receptors in colonic epithelial cells.^31^ Data from the literature showed a broad spectrum of possibilities for the therapeutic use of butyrate without having serious adverse reactions.^30,32^

**Figure 4:**
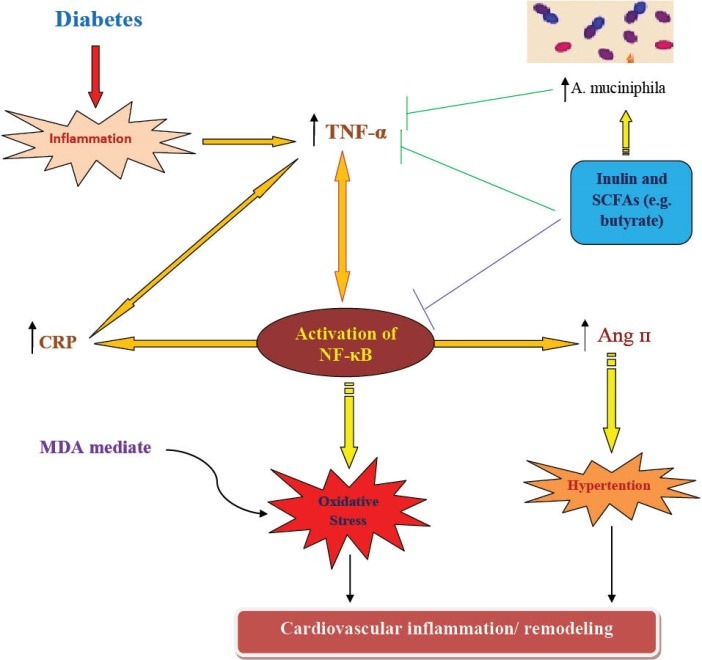
Proposed mechanisms for anti- inflammatory effects of inulin and butyrate. Prebiotics (e.g. inulin) produce metabolites such as butyrate that could promote beneficial commensally bacteria in intestinal. They can inhibit the NF-κB pathway and alleviate inflammation. NF-κB: Nuclear factor kappa- B; TNF-α: Tumor necrosis factor-alpha; Ang II: angiotensin II; CRP: C-reactive protein; MDA: malondialdehyde; SCFAs: short chain fatty acids, A. muciniphila: Akkermansia muciniphila
↑: increased level, ⊥: inhibition.

The underlying mechanisms of the effects of prebiotics and butyrate on inflammation are not yet well known but some proposed mechanisms are explained in Figure 4.

Currently, the prevalence of T2D is markedly increasing worldwide, which poses a huge burden on healthcare systems, especially in developing countries. In clinical practice, the identification of new approaches in the prevention and treatment of diabetes is a fundamental challenge.

Inflammation is the most important risk factor for CVD which is itself a major complication of T2D. A close association of gut bacterial profile and host metabolic condition has been reported previously. Recently, great attention has been paid to the *A. muciniphila* due to its ability to reduced inflammation. New complimentary supplements such as prebiotics and butyrate which are supposed to ameliorate the integrity of gut barrier and promote microbiota profile may consider new approaches to reverse the progression of chronic disease.

## Limitations of study

Our research has several limitations: we did not study some related factors in our patients such as: SCFAs in blood and stool samples and intestinal levels of endocannabinoids. Investigating the beneficial effects of NaBut for the first time in diabetic patients in the form of a randomized clinical trial was the strength of our study.

## Conclusion

The results of this study showed that inulin and butyrate exerted bifidogenic effects and ameliorated inflammatory responses in diabetic patients. However, further investigations are needed in order to confirm these positive effects.

## Competing interests

The authors declare that they have no competing interest.

## Ethical approval

The Ethics Committee of Tabriz University of Medical Sciences has approved this trial (Ethical code: IR.TBZMED.1395.778). All the participants filled out the signed consent form. This project is registered in the Iranian Registry of Clinical Trials website (identifier: IRCT201605262017N29, http://irct.ir).

## Acknowledgments

The authors thank Dr. Anari, Dr. Azizzadeh, Mr. Aghilzadeh, Mrs. Shirahmadi, Mr. Naderi Gargari, Mr. Rostamzadeh, Mr. Zargarzadeh, and all of the patients who eagerly participated in the current study. The authors also wish to thank the Research Vice Chancellor of Tabriz University of Medical Sciences for extending financial support for this study.
